# Blood Pressure Variability and Adverse Pregnancy and Cardiovascular Outcomes in the ALSPAC Cohort

**DOI:** 10.1161/JAHA.124.040547

**Published:** 2025-11-03

**Authors:** Milly Wilson, Ashwini Elanko, Hiten Mistry, Jeffrey Bone, Laura Slade, Joel Singer, Richard McManus, Peter von Dadelszen, Laura A. Magee

**Affiliations:** ^1^ Department of Women and Children’s Health, School of Life Course and Population Sciences, Faculty of Medicine King’s College London London UK; ^2^ British Columbia Children’s Hospital Research Institute University of British Columbia Vancouver Canada; ^3^ Department of Obstetrics and Gynaecology University of British Columbia Vancouver Canada; ^4^ Robinson Research Institute The University of Adelaide Adelaide South Australia Australia; ^5^ Department of Obstetrics and Gynaecology Women’s and Children’s Hospital Adelaide Australia; ^6^ School of Population and Public Health University of British Columbia Vancouver Canada; ^7^ Nuffield Department of Primary Care Health Sciences University of Oxford Oxford UK

**Keywords:** ALSPAC, blood pressure variability, cardiovascular disease, hypertension, pregnancy, preterm birth, High Blood Pressure, Preeclampsia, Hypertension

## Abstract

**Background:**

Outside pregnancy, blood pressure variability (BPV) predicts cardiovascular events. We aimed to study associations (if any) between visit‐to‐visit BPV in pregnancy and (1) adverse maternal/perinatal outcomes, and (2) long‐term maternal cardiovascular outcomes. We conducted a secondary analysis of data from ALSPAC (Avon Longitudinal Study of Parents and Children).

**Methods:**

Adjusted logistic regression assessed relationships between visit‐to‐visit BPV (by the measures of SD, average real variability, and variability independent of mean) and pregnancy outcomes (gestational/severe hypertension, preeclampsia, preterm birth, small‐for‐gestational‐age infants, neonatal intensive care unit admission, stillbirth, and perinatal death). Adjusted Cox regression assessed relationships between visit‐to‐visit BPV measures and long‐term maternal outcomes: hypertension (measured), diabetes (self‐reported), and heart disease (self‐reported) as a composite.

**Results:**

Among 12 509 women in ALSPAC, 4956 answered a follow‐up questionnaire and 4426 attended a follow‐up clinic, an average of 22 years after the index pregnancy. Measures of variability in systolic and diastolic BP (by each of SD, average real variability, and variability independent of mean) were associated with adverse pregnancy outcomes, particularly severe hypertension and preeclampsia by SD and variability independent of mean (adjusted odds ratios, 1.30–2.11). BPV in pregnancy was not associated with hypertension, diabetes, or heart disease at follow‐up in adjusted analyses.

**Conclusions:**

Our findings indicate that BP variation between antenatal visits is informative for identifying risk of short‐term adverse pregnancy outcomes, but BPV provides no long‐term utility in predicting cardiovascular risk.

**Nonstandard Abbreviations and Acronyms**
ALSPAC: Avon Longitudinal Study of Parents and Children
ARV: average real variability
BPV: blood pressure variability
HDP: hypertensive disorder of pregnancy
VIM: variability independent of the mean

**Clinical Perspective**

**What Is New**

- Increased visit‐to‐visit blood pressure variability (BPV) during pregnancy is associated with adverse pregnancy outcomes, including severe hypertension and preeclampsia, but it does not predict long‐term cardiovascular disease.

**What Are the Clinical Implications**

- Incorporating blood pressure variability assessment into routine antenatal care may enhance early risk stratification and targeted management of pregnancy complications, whereas its lack of association with long‐term cardiovascular disease outcomes suggests that other factors should guide postpregnancy cardiovascular risk evaluation.

The hypertensive disorders of pregnancy (HDPs) are associated with high rates of maternal and perinatal morbidity and mortality.^1^ As such, antepartum care is dedicated largely to the detection of hypertension, through blood pressure (BP) measurement at each antenatal care contact. An abnormal BP is ≥140/90 mm Hg, a threshold that identifies those who are at increased risk of adverse outcomes and warrant antihypertensive therapy.^2^, ^3^

There is interest in exploring whether additional characteristics of BP, other than absolute level, may improve risk stratification for adverse outcomes in pregnancy and postpartum. Systematic reviews of 23 studies in the first half of pregnancy and 12 in the second half (985 549 women in total) found no evidence that using BP thresholds lower than 140/90 mm Hg improved the identification of women and babies at higher risk of adverse outcomes.^4^, ^5^ However, systematic review of eight observational studies (138 949 women) showed that greater variation in visit‐to‐visit BP levels during pregnancy was associated with a higher risk of HDPs, preterm birth, and small‐for‐gestational age (SGA) infants.^6^

Also, beyond the implications of HDPs during pregnancy and postpartum, these conditions are associated with a heightened risk of cardiovascular disease (CVD).^7^ This relationship may be due to shared genetic and preconception lifestyle factors, HDP‐related vascular and endothelial damage during pregnancy/postpartum, or both. It is not known whether visit‐to‐visit BP variability (BPV) in pregnancy has similar associations with maternal cardiovascular risk factors and CVD. BPV outside pregnancy has such associations, including with all‐cause mortality, and the effect is over and above the effects of mean BP.^8^ As such, BPV is now included in predictive algorithms for 10‐year CVD risk in women and men.^9^

In this secondary analysis of data from ALSPAC (Avon Longitudinal Study of Parents and Children) cohort in the United Kingdom, we evaluated whether BPV in pregnancy is associated with both short‐term adverse pregnancy outcomes and long‐term maternal cardiovascular risk factors and CVD.

## METHODS

### Avon Longitudinal Study of Parents and Children

This is a secondary analysis of data from ALSPAC, a longitudinal population‐based cohort study of parents and offspring, resident in Avon, United Kingdom. ALSPAC prospectively recruited 14 541 pregnant women with an expected delivery date between April 1, 1991 and December 31, 1992.

Details of the ALSPAC cohort and study design have been published previously.^10^, ^11^ In brief, during the index pregnancy, women were sent an average of 3 questionnaires, which collected information about demographics and current health conditions. Maternity data were extracted directly from medical records. Information about long‐term outcomes was collected by a combination of questionnaires and direct evaluation at follow‐up clinics. The data collection method for each variable is summarized in Table S1.

ALSPAC study data were collected and managed using the Research Electronic Data Capture electronic data capture tools, hosted at the University of Bristol.^12^ Research Electronic Data Capture is a secure, web‐based software platform designed to support data capture for research studies. The study website contains details of all data available through a fully searchable data dictionary and variable‐of‐interest search tool.^13^

Ethical approval for the study was obtained from the ALSPAC Ethics and Law Committee and the Local Research Ethics Committees (National Health Service Haydock REC: 10/H1010/70). Informed consent for use of data was obtained from participants following the recommendations of the ALSPAC Ethics and Law Committee at the time.

### This Project

In this secondary analysis, we included all women with data on at least 3 BP measurements during pregnancy, as well as pregnancy outcomes.

Baseline maternal and pregnancy characteristics were defined as per the ALSPAC data dictionary. Social class was defined as the highest parental occupation (classes I [professional/management] to V [unskilled manual workers]).

BP values and most pregnancy outcomes were extracted from routine antenatal medical records (Table S1); if there were multiple BP measurements at an antenatal care contact, the mean was used as the reading for that visit.

Three measures of BPV were calculated: (1) SD, indicating the spread of BP measurements around each participant’s mean; (2) average real variability (ARV), a measure of the absolute successive differences between BP measurements; and (3) variability independent of the mean BP (VIM), derived from the distribution of BP values within the sample itself, making it better able to differentiate BPV from the effects of mean BP.^14^ To calculate VIM, a nonlinear model of systolic BP/diastolic BP SD against systolic BP/diastolic BP mean is fitted, estimating parameter *x*. Parameter *k* is then estimated as mean BP (sample mean BP)^x^. VIM is calculated as: *k*×SD/mean^x^.

Pregnancy outcomes were defined according to the ALSPAC protocol. Gestational hypertension was the absence of a diagnosis of hypertension outside pregnancy and at least 2 occasions of systolic BP ≥140 mm Hg or diastolic BP ≥90 mm Hg after 20 weeks’ gestation. Preeclampsia was gestational hypertension with at least 1+ (30 mg/dL) proteinuria on urine dipstick testing. Superimposed preeclampsia included women with chronic hypertension who also met the proteinuria criterion for preeclampsia. Preterm birth was birth at <37 weeks’ gestation. SGA infants were born below the 10th centile for birthweight, by sex and gestational age, according to Intergrowth‐21st standards.^15^ Stillbirth was delivery of a fetus without vital signs at ≥20 weeks’ gestation. Perinatal death combined fetal death at ≥20 weeks’ gestation and early neonatal death at <7 days after birth.

For long‐term maternal follow‐up, 2 questionnaires were completed, at 18 years after the index pregnancy and in the year 2013. Questions included whether women had been told that they had diabetes or that they had suffered a heart attack, heart failure, heart trouble, aortic aneurysm, or narrowing, with 1 or more of these taken as indicative of heart disease composite.

All women with known contact details and who had not withdrawn from the study were invited to follow‐up clinics, a mean of 18 years after the index pregnancy. At that time, clinical measurements were taken (height, weight, and BP), and it was ascertained whether women were taking hormone replacement therapy. BP was measured while women were lying down, using an Omron M6 monitor; 2 readings of systolic and diastolic BP were recorded from each arm, and the mean of these 4 readings taken as the BP for the visit. Hypertension was defined as a systolic BP ≥140 mm Hg or diastolic BP ≥90 mm Hg.

Long‐term maternal outcomes from the questionnaires and follow‐up clinics were each taken from the latest time point available. Diabetes was taken as self‐reported. Menopause was taken as self‐reported use of hormone replacement therapy at each time point, or when that measure was not available, as age >51 years.

### Statistical Analysis

Descriptive analyses were undertaken for baseline and long‐term characteristics, BPV in pregnancy, short‐term pregnancy outcomes, and long‐term maternal outcomes. Violin plots of BPV median [interquartile range] were created.

*To assess the relationship between BPV in pregnancy and pregnancy outcomes*, logistic regression was used to estimate the odds ratios (ORs) and corresponding 95% CIs. Confounders were selected a priori based on published literature linking them both to BPV and pregnancy outcomes. These included maternal age, body mass index (BMI), parity, smoking status, and use of antihypertensive medications, all of which are known to be associated with maternal cardiovascular physiology and adverse pregnancy outcomes. Mean pregnancy BP was included as a covariate, given its correlation with BPV and independent association with adverse outcomes. Continuous covariates were included as linear terms, and no interaction terms were tested in order to preserve model simplicity and interpretability. Models were fitted to both crude and adjusted data. Sensitivity analyses were conducted to address the possibility of reverse causality, whereby, a BPV‐outcome relationship could be present because the BPV is part of the outcome; SD, ARV, VIM, and mean BP were calculated following removal of BP values that were 6, 4, 2, and 1 week(s) before birth. These time periods were selected to represent increasingly conservative exclusions of late‐pregnancy BP values, based on the clinical understanding that adverse outcomes occur most commonly in the final weeks of gestation, and to be conservative, as all women are seen not less frequently than 1 to 2 weeks in late pregnancy.

*To assess the relationship between BPV in pregnancy and long‐term maternal outcomes*, Cox regression was used to estimate hazard ratios (HRs) and 95% CIs, using time since the end of each pregnancy to event or censoring as the time scale. Models were initially unadjusted, then adjusted for social class and key maternal characteristics selected a priori based on published literature documenting an association with them to both BPV and long‐term cardiovascular risk: mean BP during pregnancy, maternal age, BMI, smoking status, and parity. These are factors known to influence both short‐ and long‐term cardiovascular physiology and disease risk. Social class was included to account for socioeconomic differences that may confound both exposure and outcomes. Continuous covariates were included as linear terms, and no interaction terms were tested in order to preserve model simplicity and interpretability.

For the Cox models, the proportional hazards assumption was assessed graphically using log(−log) survival plots and was found to be reasonable. Cox regression was selected as it accommodates censoring and differing follow‐up times, allowing for appropriate estimation of hazard ratios in long‐term time‐to‐event data.

Also, to validate current understanding, we estimated the association between gestational hypertension and preeclampsia, and cardiovascular risk factors and CVD.

For all analyses, multiple imputation was used for missing data, in the multivariate imputation via chained equations package in R statistical software. Percentages of missing data and variables used in the imputation models are presented in Table S2. Fifty imputed data sets were generated, and results were pooled using Rubin’s rules. Interpretation of results was based on the magnitude of the estimated associations. A 95% CI that did not cross 1, along with a *P* value <0.05, was considered indicative of a statistically discernible association.

## RESULTS

Of 14 541 pregnancies enrolled in ALSPAC, 12 509 (86.0%) were included in our analysis, based on having at least 3 antenatal BP values and data on pregnancy outcomes; details are presented in Figure S1. Of these participants, 4956 (39.6%) answered at least 1 follow‐up questionnaire about their cardiovascular health, and 4426 (35.4%) attended at least 1 follow‐up clinic appointment. A total of 5898 individual women had some form of follow‐up data.

Pregnancy, lifestyle, and cardiovascular health characteristics, for the pregnancy cohort and then those who participated in follow‐up, are presented in Table 1. Women in pregnancy were just under 30 years of age, and primarily (89%) of White race. BMI was within the normal range (22.2 kg/m^2^). Just under half (43.5%) were nulliparous, and many (26.6%) had active cigarette use. Some drank alcohol during pregnancy (15.2%), and few took calcium (3.3%), aspirin (<1%), or antihypertensives (types of which were unknown) (<1%). Fewer than 5% reported diabetes or chronic kidney disease. Characteristics of those who participated in follow‐up were similar, with the exception that they were more often White (96.4%) and less often smokers (16.8%).

**Table 1:** Pregnancy Characteristics

		In pregnancy*	Follow‐up†
Total N (%)		12 509	4956
Maternal pregnancy characteristics			
Age, y		28.0 [24.0–31.0]	29.0 [26.0–32.0]
Race	White	11 122 (88.9)	4779 (96.4)
	Black	124 (1.0)	26 (0.5)
	Asian	102 (0.8)	32 (0.6)
	Other	73 (0.6)	33 (0.7)
Body mass index (kg/m^2^)		22.2 [20.5–24.4]	22.0 [20.5–24.1]
Nulliparous		5436 (43.5)	2336 (47.1)
Cigarette use		3331 (26.6)	832 (16.8)
Alcohol		1901 (15.2)	764 (15.4)
Calcium		419 (3.3)	172 (3.5)
Aspirin		81 (0.6)	33 (0.7)
Education	Certificate of secondary education /none	2306 (18.4)	496 (10.0)
	Vocational	1144 (9.1)	358 (7.2)
	O‐level	3986 (31.9)	1690 (34.1)
	A‐level	2574 (20.6)	1373 (27.7)
	Degree	1477 (11.8)	960 (19.3)
Social class	Professional	549 (4.4)	363 (7.3)
	Managerial	2942 (23.5)	1585 (32.0)
	Skilled (nonmanual)	3987 (31.9)	1787 (36.1)
	Skilled (manual)	738 (5.9)	241 (4.9)
	Partly skilled	917 (7.3)	278 (5.6)
	Unskilled	208 (1.7)	59 (1.2)
Diabetes		118 (0.9)	44 (0.9)
Chronic kidney disease		514 (4.1)	184 (3.7)
BP measurements during pregnancy			
No, measurements	<20 weeks’ gestation	3.0 [2.0–3.0]	3.0 [2.0–3.0]
	≥20 week’s gestation	11.0 [9.0–13.0]	11.0 [9.0–12.0]
Systolic BP mm Hg	Overall	114.1 [108.8–119.7]	114.0 [108.6–119.6]
	<20 weeks’ gestation	110.0 [105.0–118.3]	110.0 [105.0–118.3]
	≥20 week’s gestation	114.7 [109.3–120.5]	114.6 [109.1–120.4]
Diastolic BP mm Hg	Overall	67.5 [63.8–71.6]	67.5 [63.9–71.7]
	<20 weeks’ gestation	65.0 [60.0–70.0]	65.0 [60.0–70.0]
	≥20 week’s gestation	68.1 [64.2–72.5]	68.2 [64.3–72.5]
BPV (systolic)	SD	9.2 [7.6–11.1]	9.1 [7.5–11.0]
	ARV	8.8 [6.8–10.9]	8.7 [6.7–10.8]
	VIM	9.4 [7.7–11.2]	9.3 [7.7–11.1]
BPV (diastolic)	SD	7.3 [5.9–8.8]	7.1 [5.8–8.7]
	ARV	6.4 [5.0–8.1]	6.4 [4.8–8.0]
	VIM	7.5 [6.1–9.0]	7.3 [6.0–9.0]
Maternal outcomes			
Chronic hypertension		572 (4.6)	224 (4.5)
Gestational hypertension		1874 (15.0)	737 (14.9)
Preeclampsia		316 (2.5)	111 (2.2)
Antihypertensives (at any point in pregnancy)		51 (0.4)	17 (0.3)
Severe hypertension (regardless of hypertension type)		761 (6.1)	281 (5.7)
Gestational diabetes		56 (0.4)	19 (0.4)
Method of delivery	Spontaneous	9399 (75.1)	3664 (73.9)
	Breech	149 (1.2)	53 (1.1)
	Cesarean	1376 (11.0)	523 (10.6)
	Instrumental vaginal	1516 (12.1)	692 (14.0)
Perinatal outcomes			
Stillbirth		38 (3.0/1000)	<5 (<1.0/1000)
Neonatal death		73 (5.8/1000)	<5 (<1.0/1000)
Gestational age at birth, wks		40.0 [39.0–41.0]	40.0 [39.0–41.0]
Preterm birth <37 wks’		752 (6.0)	258 (5.2)
Birthweight, g		3420 [3100–3750]	3450 [3140–3760]
SGA‐infant		1174 (9.4)	400 (8.1)
Special care baby unit admission		756 (6.0)	294 (5.9)

Median [interquartile range] or N (%) unless otherwise stated. ARV indicates average real variability; BP, blood pressure; BPV, blood pressure variability; SGA, small‐for‐gestational age; and VIM, variability independent of mean.

* Characteristics of the entire pregnancy cohort.

^†^ Characteristics of women from the pregnancy cohort who also answered at least 1 follow‐up postpregnancy questionnaire.

BP was measured a median of 13 times during pregnancy, most often from 20 weeks’ gestation (Table 1). Median BP was 114.1/67.5 mm Hg, with both systolic BP and diastolic BP having higher values in the second (versus first) half of pregnancy. BPV was slightly higher for systolic than diastolic BP, with similar values across the BPV measures; these are presented graphically in Figure. BPV did not differ among the subgroup of women participating in long‐term follow‐up. BP and BPV measures were similar for the cohort overall and those who participated in follow‐up.

**Figure 1:**
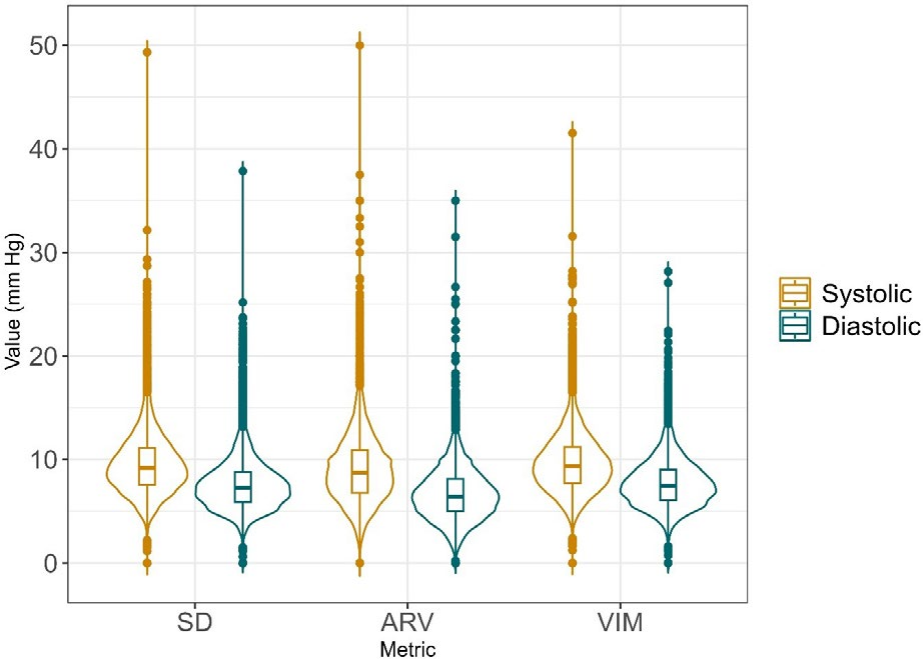
Violin plots for BPV metrics. Data are median [interquartile range]. ARV indicates average real variability; BPV, blood pressure variability; and VIM, variability independent of the mean.

Regarding pregnancy outcomes, gestational hypertension occurred frequently (15%), far more often than did preeclampsia (2.5%) (Table 1). A number of women (6.1%) developed severe hypertension, but gestational diabetes was rare (<1%). Most women (75.1%) had spontaneous vaginal births and at a median of 40.0 weeks. Small proportions of babies were born preterm (6.0%) or SGA (9.4%), or required special care baby unit admission (6.0%). The subgroup of women who participated in follow‐up appeared less likely to have suffered a stillbirth or neonatal death and may have been slightly less likely to have had a baby born preterm (5.2%) or SGA (8.1%).

### BPV and Pregnancy Outcomes

Table 2 shows the adjusted and unadjusted ORs for BPV measures and pregnancy outcomes, for systolic and diastolic BP. Higher systolic and diastolic BPV was consistently associated with adverse pregnancy outcomes, with the exceptions of stillbirth and neonatal death. The association between BPV and adverse pregnancy outcomes was particularly strong for the BPV measures of SD and VIM, and the outcome of severe hypertension and preeclampsia (adjusted ORs, 1.30–2.11).

**Table 2:** Crude and Adjusted Odds Ratios for BPV and Pregnancy Outcomes*

Outcomes		Systolic BP (95% CI)	Wald’s test (*P* value)	Diastolic BP (95% CI)	Wald’s test (*P* value)
Gestational hypertension					
SD	Crude	1.39 (1.37–1.42)	<0.001	1.49 (1.45–1.52)	<0.001
	Adjusted	1.19 (1.14–1.23)	<0.001	1.17 (1.12–1.22)	<0.001
ARV	Crude	1.15 (1.13–1.16)	<0.001	1.13 (1.11–1.15)	<0.001
	Adjusted	1.09 (1.07–1.12)	<0.001	1.07 (1.04–1.10)	<0.001
VIM	Crude	1.17 (1.15–1.19)	<0.001	1.17 (1.15–1.19)	<0.001
	Adjusted	1.35 (1.32–1.39)	<0.001	1.37 (1.33–1.41)	<0.001
Severe hypertension					
SD	Crude	1.86 (1.80–1.93)	<0.001	1.69 (1.64–1.74)	<0.001
	Adjusted	1.98 (1.88–2.09)	<0.001	1.42 (1.37–1.47)	<0.001
ARV	Crude	1.27 (1.24–1.29)	<0.001	1.19 (1.16–1.22)	<0.001
	Adjusted	1.27 (1.24–1.31)	<0.001	1.15 (1.11–1.19)	<0.001
VIM	Crude	1.46 (1.42–1.49)	<0.001	1.46 (1.42–1.49)	<0.001
	Adjusted	2.11 (1.98–2.24)	<0.001	1.49 (1.43–1.55)	<0.001
Preeclampsia					
SD	Crude	1.45 (1.41–1.49)	<0.001	1.69 (1.63–1.76)	<0.001
	Adjusted	1.30 (1.26–1.35)	<0.001	1.44 (1.37–1.50)	<0.001
ARV	Crude	1.13 (1.11–1.16)	<0.001	1.19 (1.15–1.24)	<0.001
	Adjusted	1.04 (1.01–1.07)	0.024	1.10 (1.05–1.15)	<0.001
VIM	Crude	1.32 (1.28–1.36)	<0.001	1.32 (1.28–1.36)	<0.001
	Adjusted	1.33 (1.27–1.38)	<0.001	1.54 (1.45–1.63)	<0.001
Preterm birth					
SD	Crude	1.13 (1.11–1.16)	<0.001	1.20 (1.17–1.23)	<0.001
	Adjusted	1.12 (1.09–1.14)	<0.001	1.17 (1.14–1.20)	<0.001
ARV	Crude	1.07 (1.04–1.09)	<0.001	1.12 (1.10–1.15)	<0.001
	Adjusted	1.05 (1.03–1.07)	<0.001	1.11 (1.08–1.14)	<0.001
VIM	Crude	1.11 (1.09–1.14)	<0.001	1.11 (1.09–1.14)	<0.001
	Adjusted	1.11 (1.09–1.14)	<0.001	1.18 (1.14–1.22)	<0.001
SGA infant					
SD	Crude	1.07 (1.05–1.09)	<0.001	1.10 (1.08–1.13)	<0.001
	Adjusted	1.07 (1.04–1.09)	<0.001	1.07 (1.05–1.10)	<0.001
ARV	Crude	1.03 (1.02–1.05)	<0.001	1.03 (1.01–1.06)	0.004
	Adjusted	1.03 (1.01–1.05)	<0.001	1.02 (1.00–1.05)	0.075
VIM	Crude	1.07 (1.05–1.09)	<0.001	1.07 (1.05–1.09)	<0.001
	Adjusted	1.06 (1.04–1.09)	<0.001	1.08 (1.05–1.11)	<0.001
Neonatal intensive care unit admission					
SD	Crude	1.13 (1.11–1.16)	<0.001	1.15 (1.12–1.18)	<0.001
	Adjusted	1.12 (1.09–1.14)	<0.001	1.11 (1.08–1.14)	<0.001
ARV	Crude	1.07 (1.05–1.09)	<0.001	1.08 (1.05–1.11)	<0.001
	Adjusted	1.06 (1.04–1.08)	<0.001	1.06 (1.04–1.09)	<0.001
VIM	Crude	1.11 (1.09–1.14)	<0.001	1.11 (1.09–1.14)	<0.001
	Adjusted	1.11 (1.08–1.14)	<0.001	1.10 (1.06–1.13)	<0.001
Stillbirth					
SD	Crude	1.11 (1.03–1.20)	0.007	1.06 (0.95–1.19)	0.280
	Adjusted	1.07 (0.93–1.23)	0.357	0.98 (0.80–1.21)	0.878
ARV	Crude	1.07 (0.98–1.15)	0.119	1.09 (0.98–1.21)	0.096
	Adjusted	1.05 (0.92–1.19)	0.488	0.96 (0.80–1.15)	0.640
VIM	Crude	1.13 (1.04–1.23)	0.005	1.13 (1.04–1.23)	0.005
	Adjusted	1.07 (0.93–1.23)	0.375	1.00 (0.82–1.21)	0.990
Neonatal death					
SD	Crude	1.09 (1.03–1.16)	0.004	1.05 (0.96–1.14)	0.305
	Adjusted	1.09 (0.99–1.20)	0.071	1.05 (0.92–1.20)	0.474
ARV	Crude	1.07 (1.01–1.13)	0.028	1.08 (0.99–1.16)	0.071
	Adjusted	1.08 (1.00–1.17)	0.051	1.00 (0.89–1.14)	0.923
VIM	Crude	1.11 (1.04–1.19)	0.001	1.11 (1.04–1.19)	0.001
	Adjusted	1.08 (0.98–1.20)	0.106	1.05 (0.91–1.20)	0.523

Data are crude and adjusted OR (95% CI). Adjusted ORs are adjusted for maternal age, maternal body mass index, parity, and use of antihypertensives. ARV indicates average real variability; BP, blood pressure; BPV, blood pressure variability; SGA, small‐for‐gestational age; OR, odds ratio; and VIM, variability independent of mean.

* Cells in which 95% CIa do not overlap 1.0 are highlighted in green.

In adjusted sensitivity analyses removing BP values up to 6 weeks before birth (Table S3), the association between BPV and either gestational hypertension or preeclampsia was unchanged, but the associations with adverse outcomes for the baby were attenuated, and progressively included the null by the analysis restricted to BPV from BP measurements taken no later than 6 weeks before birth.

### BPV and Cardiovascular Outcomes

Characteristics of the women who participated in follow‐up, at the time of that follow‐up, are presented in Table 3. Women were followed up just over 20 years after pregnancy, at a median age of 50 years. Median BMI was at the lower boundary of the overweight range (ie, 25 kg/m^2^). Just over 10% had active cigarette use. Around 12% were menopausal when assessed by questionnaire; this was slightly lower (9%) when assessed at a follow‐up clinic visit. At the face‐to‐face visits, BP was on average 117.5/70.5 mm Hg, with 15.5% of women having a systolic BP of ≥140 mm Hg or diastolic BP ≥90 mm Hg. Few women (<3%) reported having been told they had diabetes. Of those who answered a questionnaire, 4.4% experienced the heart disease composite outcome.

**Table 3:** Follow‐Up Characteristics and Outcomes

	Follow‐up (questionnaires)*	Follow‐up (clinics)†
Y postpartum	22.0 [21.0–22.0]	22.0 [20.0–23.0]
Long‐term covariates		
Current age at follow‐up	50.0 [46.0–53.0]	51.0 [47.0–54.0]
Current body mass index at follow‐up	25.2 [22.6–28.6]	25.8 [23.0–29.5]
Current cigarette use at follow‐up	534 (10.8)	**…**
Use of hormone replacement therapy or age >51 y	581 (11.7)	440 (9.9)
Cardiovascular risk factors at follow‐up		
Antihypertensives	**…**	248 (5.6)
BP (mm Hg)		
Systolic	**…**	117.5 [109.5–127.5]
Diastolic	**…**	70.5 [64.5–77.0]
Elevated BP	**…**	686 (15.5)
Diabetes	142 (2.9)	**…**
CVD by self‐report at follow‐up		
Heart attack	34 (0.7)	**…**
Heart failure	20 (0.4)	**…**
Aortic aneurysm	18 (0.4)	**…**
Narrowing or hardening of arteries	18 (0.4)	**…**
Stroke	49 (1.0)	**…**
Heart disease composite (one/more of CVD by self‐report, as above)	216 (4.4)	**…**

**…** indicates that information was not available; BP, blood pressure; CVD, cardiovascular disease; and FOM, follow‐up mothers.

* Characteristics of women from the pregnancy cohort who also answered either the V or T follow‐up postpregnancy questionnaire.

^†^ Characteristics of women from the pregnancy cohort who also attended 1 or more of FOM1, FOM2, FOM3, or FOM4.

Women who had gestational hypertension or preeclampsia in their ALSPAC pregnancy (versus those who did not), had higher adjusted risk of hypertension and the self‐reported heart disease composite outcome (Table 4); the results were similar in unadjusted and adjusted analyses. Gestational hypertension or preeclampsia exposure conferred a higher risk of diabetes only in unadjusted (and not adjusted) analysis.

**Table 4:** Crude and Adjusted Estimates for Relationship Between Gestational Hypertension/Preeclampsia and CVD

	Gestational hypertension/preeclampsia	
	HR (95% CI)	*P* value
Follow‐up clinic visit measurement		
Hypertension		
Crude	2.26 (1.92–2.67)	<0.001
Adjusted	2.34 (1.94–2.83)	<0.001
Self‐reported measures		
Diabetes		
Crude	3.12 (2.22–4.39)	<0.001
Adjusted	2.19 (1.45–3.31)	<0.001
Heart disease composite*		
Crude	1.47 (1.07–2.02)	0.018
Adjusted	1.78 (1.24–2.55)	0.002

Adjustments for social class, and pregnancy factors: age, body mass index, smoking, and parity. CVD indicates cardiovascular disease; and HR, hazard ratio.

* Heart attack, heart failure, aortic aneurysm, narrowing, and other heart trouble.

Table 5 shows that in unadjusted, but not adjusted, analyses, higher BPV (by SD and ARV) was associated with hypertension and diabetes. However, there was no association between BPV and the heart disease composite outcome in unadjusted or adjusted analyses.

**Table 5:** Crude and Adjusted Estimates for Relationship Between BPV and CVD

		Systolic BPV		Diastolic BPV	
		HR (95% CI)	*P* value	HR (95% CI)	*P* value
Clinic measurement					
Hypertension					
SD	Crude	1.05 (1.03–1.08)	<0.001	1.10 (1.07–1.13)	<0.001
	Adjusted	1.02 (0.99–1.05)	0.256	1.00 (0.97–1.03)	0.974
ARV	Crude	1.03 (1.01–1.05)	0.012	1.05 (1.02–1.08)	<0.001
	Adjusted	1.00 (0.97–1.03)	1.000	1.01 (0.98–1.04)	0.479
VIM	Crude	0.99 (0.97–1.02)	0.611	1.03 (1.00–1.07)	0.068
	Adjusted	1.02 (0.99–1.05)	0.246	1.00 (0.97–1.04)	0.818
Self‐reported measures					
Diabetes					
SD	Crude	1.08 (1.03–1.14)	0.002	1.10 (1.04–1.17)	0.001
	Adjusted	1.02 (1.00–1.04)	0.097	1.04 (0.97–1.12)	0.301
ARV	Crude	1.05 (1.00–1.10)	0.033	1.06 (1.00–1.13)	0.073
	Adjusted	1.04 (0.98–1.09)	0.187	1.03 (0.96–1.12)	0.354
VIM	Crude	1.02 (0.97–1.08)	0.453	1.04 (0.96–1.11)	0.334
	Adjusted	1.02 (0.97–1.07)	0.403	1.04 (0.96–1.12)	0.384
Heart disease composite*		HR (95% CI)	*P* value	HR (95% CI)	*P* value
SD	Crude	0.99 (0.95–1.04)	0.650	1.03 (0.98–1.08)	0.295
	Adjusted	0.99 (0.94–1.04)	0.680	0.98 (0.93–1.05)	0.599
ARV	Crude	1.00 (0.96–1.05)	0.878	1.03 (0.98–1.09)	0.242
	Adjusted	1.00 (0.96–1.05)	0.861	1.00 (0.94–1.06)	0.947
VIM	Crude	0.95 (0.91–1.00)	0.063	1.01 (0.95–1.07)	0.732
	Adjusted	0.99 (0.93–1.04)	0.594	0.99 (0.93–1.05)	0.674

* Heart attack, heart failure, aortic aneurysm, narrowing, and other heart trouble.

Adjustments for social class, and pregnancy factors: mean blood pressure, age, body mass index, smoking, and parity. ARV indicates average real variability; BPV, blood pressure variability;HR, hazard ratio; and VIM, variation independent of mean.

This study was conducted and reported in accordance with the Strengthening the Reporting of Observational Studies in Epidemiology guidelines for cohort studies.

## DISCUSSION

This secondary analysis of ALSPAC cohort data demonstrated associations between higher BPV and adverse pregnancy outcomes, with the exception of stillbirth and neonatal death. The association was particularly strong for the BPV measures of SD and VIM, and the outcomes of severe hypertension and preeclampsia. These findings were consistent when BP measurements up to 6 weeks before delivery were excluded from calculation of BPV, particularly for maternal outcomes, which provided little evidence of reverse causality.

Although associations were evident between gestational hypertension/preeclampsia in the index ALSPAC pregnancy, and hypertension, diabetes, and heart disease at long‐term follow‐up, there was no relationship between BPV in pregnancy and cardiovascular risk factors or CVD in the long term.

### Comparison With the Literature

These findings corroborate those of our recent systematic review, in which six studies from diverse socioeconomic settings, quantified positive associations between higher BPV (by SD, ARV, or coefficient of variation) and more adverse pregnancy outcomes, independent of mean BP, and in normotensive, hypertensive, and unselected pregnancies.^6^ Three studies have also examined the impact of excluding from calculation of BPV, BP measurements closest to delivery; each of these studies concurred that although effect estimates for the BPV‐outcome relationship were attenuated, significant associations persisted between higher BPV and more hypertension or preeclampsia.^16^, ^17^, ^18^ Outside pregnancy, long‐term (visit‐to‐visit) BPV may be a perpetrator or result of arterial stiffness, or reflect poor BP control, inconsistent office measurement, or medication adherence.^19^ In pregnancy, BPV may be on the same pathogenic pathway as gestational hypertension and preeclampsia, a result of interplay between uteroplacental development, angiogenic imbalance, and endothelial dysfunction.^1^ BPV could also be the impact of adverse cardiac remodeling to hypertension or preeclampsia in a previous pregnancy.^20^

With regard to the link between pregnancy hypertension and CVD, consistent with a robust body of research,^21^, ^22^, ^23^ we did observe in this ALSPAC cohort that a history of gestational hypertension or preeclampsia was associated with an elevated long‐term risk of developing hypertension, diabetes, and a composite of heart disease. However, we did not observe a relationship between higher BPV in pregnancy and development of long‐term cardiovascular outcomes, in measures that were self‐reported by women (i.e., diabetes and the heart disease composite) or assessed in clinic directly (ie, hypertension). We are unaware of existing literature on this topic. However, our observed lack of an association between BPV and cardiovascular outcomes may be related to the shorter time span over which BPV in pregnancy is measured (ie, 9 months), in comparison to the lengthy follow‐up periods of studies exploring BPV and CVD outside pregnancy (ie, 24 months to 5 years).^24^, ^25^, ^26^, ^27^ Alternatively, higher BPV in pregnancy may not reflect substantial arterial stiffening or cardiovascular deregulation, but a promptly resolving phenomenon driven by uteroplacental mismatch, and posing no long‐term, independent risk to maternal cardiovascular health. Furthermore, despite our crude analyses demonstrating some associations between BPV and long‐term cardiovascular outcomes, these associations were attenuated after adjusting for traditional cardiovascular risk factors including mean BP, age, BMI, smoking, and parity. This attenuation may imply that established cardiovascular risk factors play a more dominant role in determining long‐term cardiovascular health than BPV in pregnancy, limiting its utility as an independent risk stratification tool. Although BPV is associated with HDPs, which in turn are strong predictors of future CVD, BPV itself may not have a direct causal role in long‐term cardiovascular risk. Instead, BPV could serve as an early marker of underlying vascular dysfunction that contributes to HDPs but does not independently predict future CVD once HDPs and other risk factors are accounted for. This may explain the observed disconnect, whereby BPV is linked to HDPs, and HDPs are linked to CVD, yet BPV does not directly correlate with long‐term cardiovascular outcomes.

Outside pregnancy, extensive research from both observational studies and clinical trials consistently demonstrates that long‐term fluctuations in BP between visits play a crucial role in enhancing the risk of CVD.^8^ This body of evidence is robust and has prompted risk assessment tools (QRISK‐3) to include the SD of systolic BP in CVD risk evaluations.^9^ Although guidelines have emphasized the significance of BPV in understanding patient profiles,^28^ it is not clear that reducing BPV will reduce cardiovascular risk.

### Research Implications

Incorporating BPV measurement into antenatal screening may improve assessment of maternal and perinatal risk and inform surveillance. However, future work will be required. First, BPV must be tested as a repeated measure, mimicking the current use of overall BP level, now that this and previous research has confirmed the association between BPV and adverse pregnancy outcomes, through calculation of BPV retrospectively, as one BPV value per pregnancy. Second, this is complicated by varying antenatal visit intervals and frequencies, the impact of which on BPV has not been ascertained. Third, determining a BPV threshold and a target value for intervention would be important. Fourth, the potential role of antihypertensive therapy should be considered; outside pregnancy, long‐acting calcium channel blockers are associated with lower BPV^29^; but whether this is true in pregnancy (for the commonly used nifedipine) should be established. Importantly, few other antihypertensive classes of drugs have an impact on BPV, which appears to be primarily attributed to reductions in arterial compliance.^30^ Finally, future work could determine whether individual BPV during pregnancy is correlated with BPV (and consequent cardiovascular risk) beyond pregnancy. In this analysis, limited BP measurements at follow‐up prevented a direct comparison with the more frequent measurements taken during pregnancy.

### Strengths and Limitations

Strengths of our analysis include application of the VIM metric for BPV calculation, testing its validity in pregnancy where it is insofar published, and comparing it to more traditional BPV metrics (ie, SD and ARV). We reported pregnancy outcomes and long‐term cardiovascular outcomes in the same cohort, with a long (>20‐year) duration of maternal follow‐up. We confirmed the anticipated association between gestational hypertension and preeclampsia and cardiovascular outcomes, Finally, our sample size was large.

There are limitations of our analysis. ALSPAC is a primarily White cohort from whom the findings may not be generalizable to more ethnically diverse populations. Other than parity at long‐term follow‐up, no information was collected about intercurrent pregnancies or associated complications. BP data were extracted from medical records by the ALSPAC team and not measured using standardized technique, as in a research clinic, although this should make our findings more generalizable. Additionally, the calculation of BPV may be influenced by the nonrandom physiological changes in BP across pregnancy. Because VIM can be derived from as few as 2 data points, it is possible that BPV measures are exaggerated in individuals experiencing the most pronounced net BP increase during pregnancy, particularly given the relatively sparse BP readings per woman in pregnancy. Although we adjusted for mean BP to account for overall BP changes, we cannot fully exclude the possibility that BPV is partly capturing the trajectory of BP rise rather than true short‐term, visit‐to‐visit variability. Future studies with more frequent BP measurements across gestation could help clarify whether BPV independently predicts pregnancy outcomes beyond the net BP increase itself. We modeled continuous covariates (such as maternal age and BMI) as linear predictors, which may not have captured nonlinear relationships with the outcomes; this was done to preserve model simplicity and interpretability, but we acknowledge that is may lead to some residual confounding or bias. The data used were from the early 1990s, and although changes have occurred in antenatal care frequency and nature (eg, prenatal diagnosis), BP is still measured at each visit, and if anything, women in ALSPAC received more antenatal care visits than prescribed by the current National Institute for Health and Care Excellence guidelines.^31^ We do not have the particulars of the antihypertensives used or medication adherence; however, the latter tends to be high in pregnancy and the antihypertensives most likely to be prescribed in the 1990s were methyldopa and labetalol, for which the impact on BPV in pregnancy is not yet known. The attrition in postpregnancy follow‐up may include cardiac fatalities, the potential impact of which on relationships cannot be assessed. The age of follow‐up for women (~50 years), whereas more than 20 years from the ALSPAC pregnancy, is still when most women are pre‐ or perimenopausal, so CVD events are still very uncommon. Cardiovascular outcomes were obtained by self‐report and thus may not have been accurately assessed.

## CONCLUSIONS

Our results indicate that higher variation in BP between antenatal visits is an accessible, informative measure that is associated with enhanced risk of adverse pregnancy outcomes and warrants future evaluation in prospective studies. However, we found no evidence that BPV in pregnancy provides long‐term utility in predicting future maternal CVD risk.

## Sources of Funding

The UK Medical Research Council and Wellcome (Grant ref: 217065/Z/19/Z) and the University of Bristol provide core support for ALSPAC. This publication is the work of the authors and Milly Wilson, Ashwini Elanko, Jeffrey Bone, Laura Slade, Hiten Mistry, Joel Singer, Richard McManus, Peter von Dadelszen, and Laura Magee will serve as guarantors for the contents of this paper. A comprehensive list of grants funding is available on the ALSPAC website: (https://www.bristol.ac.uk/alspac/external/documents/grant‐acknowledgements.pdf); This research was specifically funded by The British Heart Foundation (SP/07/008/24066), The Wellcome Trust (WT092830/Z/10/Z) and The Lifelong Health and Wellbeing (LLHW) via the MRC (G1001357). Richard McManus acknowledges funding from yhe National Institute for Health and Care Research Oxford and Thames Valley Applied Research Consortium and is a National Institute for Health and Care Research Senior Investigator.

## Disclosures

None.

## Supporting information

Tables S1–S3
Figure S1
