# Supplementary material for: Blood Pressure Variability and Adverse Pregnancy and Cardiovascular Outcomes in the ALSPAC Cohort
Source: J Am Heart Assoc. 2025 Nov 3;14(21):e040547. doi: 10.1161/JAHA.124.040547 (PMC12684795; doi:10.1161/JAHA.124.040547)
Supplement: Supplementary file 1 — Tables S1–S3 Figure S1 [file JAH3-14-e040547-s001.pdf]

SUPPLEMENTAL MATERIAL

**Table S1.** Data collection method for baseline maternal and pregnancy characteristics, pregnancy outcomes, and long-term maternal outcomes			
**Characteristics**	**Maternal report by relevant questionnaire**	**From maternity records**	**From follow-up clinics**
**Demographics**			
Maternal age	●	-	NA
Ethnicity	●	-	NA
Education	●	-	NA
Social class	●	-	NA
BMI (kg/m2)	●	-	NA
Chronic hypertension	-	●	NA
Pre-gestational DM	●	-	NA
Chronic kidney disease	●	-	NA
Prior GDM	●	-	NA
**Pregnancy characteristics & outcomes**			
Nulliparous	●	-	NA
Cigarette use in pregnancy	●	-	NA
Alcohol in pregnancy	●	-	NA
Took supplemental calcium	●	-	NA
Took low-dose aspirin	●	-	NA
BP values	-	●	NA
Use of antihypertensives	●	-	NA
Stillbirth or neonatal death	-	●	NA
Gestational age at birth	-	●	NA
Birthweight	-	●	NA
Mode of delivery	-	●	NA
NICU admission	●	-	NA
**Long-term outcomes**			
CV risk factors			
Hypertension	-	NA	●
Using antihypertensives	-	NA	●
Diabetes	●	NA	●
CVD			
Heart disease composite (one/more of)	●	NA	-
Heart attack	●	NA	-
Heart failure	●	NA	-
Aortic aneurysm	●	NA	-
Narrowing/hardening of arteries	●	NA	-
Stroke	●	NA	-
Covariates at follow-up			
Age	●	NA	●
Ethnicity	●	NA	-
Social class	●	NA	-
BMI	●	NA	●
Smoker	●	NA	-

**Table S1.** Data collection method for baseline maternal and pregnancy characteristics, pregnancy outcomes, and long-term maternal outcomes			
**Characteristics**	**Maternal report by relevant questionnaire**	**From maternity records**	**From follow-up clinics**
Hormone replacement therapy	●	NA	●
Parity	●	NA	-

BMI indicates body mass index; cIMT, carotid intima media thickness; CV, cardiovascular; CVD, cardiovascular disease; GDM, gestational diabetes mellitus; NA, not applicable; NICU, neonatal intensive care unit.

**Table S2.** N (%) Missing data in maternal and pregnancy characteristics, pregnancy outcomes, and long-term maternal outcomes (N (%))				
		**In pregnancy**	**Follow-up (questionnaires)**	**Follow-up (clinics)**
Of total N (%)		12,509	4956	4426
**Maternal & Pregnancy Characteristics**				
Age*†		528 (4.2)	82 (1.7)	93 (2.1)
Ethnicity	White	1088 (8.7)	86 (1.7)	121 (2.7)
	Black			
	Asian			
	Other			
BMI (kg/m^2)*†		1816 (14.5)	359 (7.2)	344 (7.8)
Nulliparous*†		696 (5.6)	158 (3.2)	151 (3.4)
Smoking*†		103 (0.8)	7 (0.1)	9 (0.2)
Alcohol		449 (3.6)	71 (1.4)	54 (1.2)
Calcium		448 (3.6)	76 (1.5)	63 (1.4)
Aspirin		399 (3.2)	64 (1.3)	50 (1.1)
Education	CSE/none	1022 (8.2)	79 (1.6)	108 (2.4)
	Vocational			
	O-Level			
	A-Level			
	Degree			
Social class*†	Professional	3168 (25.3)	643 (13.0)	615 (13.9)
	Managerial			
	Skilled (non-manual)			
	Skilled (manual)			
	Partly skilled			
	Unskilled			
Previous hypertension		1200 (9.6)	181 (3.7)	188 (4.2)
Diabetes		1010 (8.1)	99 (2.0)	116 (2.6)
CKD		964 (7.7)	90 (1.8)	104 (2.3)
Antihypertensives (at any point in pregnancy)		0 (0)	0 (0)	0 (0)
**Maternal Outcomes**				
Chronic hypertension		464 (3.7)	142 (2.9)	118 (2.7)
Gestational hypertension		285 (2.3)	97 (2.0)	86 (1.9)
Preeclampsia		316 (2.5)	0 (0)	0 (0)
Severe hypertension (any hypertension type)		0 (0)	0 (0)	0 (0)
Gestational diabetes		964 (7.7)	90 (1.8)	104 (2.3)
Method of delivery	Spontaneous	69 (0.6)	24 (0.5)	17 (0.4)
	Breech			
	Caesarean			

Table S2. N (%) Missing data in maternal and pregnancy characteristics, pregnancy outcomes, and long-term maternal outcomes (N (%))

	**In pregnancy**	**Follow-up (questionnaires)**	**Follow-up (clinics)**
Of total N (%)	12,509	4956	4426
Instrumental vaginal			
**Perinatal outcomes**			
Stillbirth	0 (0)	0 (0)	0 (0)
Neonatal death	0 (0)	0 (0)	0 (0)
Gestational age at birth	0 (0)	0 (0)	0 (0)
PTB <37 weeks’	0 (0)	0 (0)	0 (0)
Birthweight (g)	115 (0.9)	34 (0.7)	30 (0.7)
SGA	178 (1.4)	60 (1.2)	53 (1.2)
NICU admission	1285 (10.3)	130 (2.6)	164 (3.7)
**Long term covariates**			
Current age at f/u	-	0 (0)	0 (0)
Current BMI at f/u	-	762 (15.4)	11 (0.2)
Current smoker at f/u	-	68 (1.4)	-
Use of HRT or age >51	-	0 (0)	0 (0)
**CV risk factors at f/u**			
Antihypertensives	-	-	244 (5.5)
Blood pressure (mmHg)			
Systolic	-	-	54 (1.2)
Diastolic	-	-	54 (1.2)
Hypertension	-	-	54 (1.2)
Diabetes mellitus	-	31 (0.6)	92 (2.1)
**CVD by self-report at f/u**			
Heart attack	-	41 (0.8)	-
Heart failure	-	42 (0.8)	-
Aortic aneurysm	-	53 (1.1)	-
Narrowing or hardening of arteries	-	48 (1.0)	-
Stroke	-	28 (0.6)	-
Heart disease composite	-	69 (1.4)	-

BMI indicates body mass index; CKD, chronic kidney disease; CVD, cardiovascular disease; f/u, follow-up; HRT, hormone replacement therapy; NICU, neonatal intensive care unit; PTB, preterm birth; SGA, small-for-gestational-age.

* Variables used in the imputation model for pregnancy outcomes analysis.

† Variables used in the imputation model for maternal follow-up analysis.

**Table S3.** Sensitivity Analyses: Adjusted ORs For Relationship Between BPV and Pregnancy Outcomes, Excluding BP Values One-, Two-, Four-, and Six- Weeks from Birth									
**Outcomes**	**Systolic BP**	**Wald's Test (p)**	**Diastolic BP**	**Wald's Test (p)**	**Outcomes**	**Systolic BP**	**Wald's Test (p)**	**Diastolic BP**	**Wald's Test (p)**
**One- Week Sensitivity Analysis**					**Two- Week Sensitivity Analysis**				
**Gestational hypertension**					**Gestational hypertension**				
SD	1.16 (1.13,1.18)	<0.001	1.15 (1.12,1.18)	<0.001	*SD*	1.14 (1.11,1.17)	<0.001	1.14 (1.10,1.17)	<0.001
ARV	1.08 (1.06,1.09)	<0.001	1.06 (1.03,1.08)	<0.001	*ARV*	1.07 (1.05,1.08)	<0.001	1.05 (1.02,1.07)	<0.001
VIM	1.16 (1.13,1.18)	<0.001	1.16 (1.23,1.19)	<0.001	*VIM*	1.14 (1.11,1.16)	<0.001	1.13 (1.10,1.16)	<0.001
**Severe hypertension**					**Severe hypertension**				
SD	1.39 (1.35,1.44)	<0.001	1.23 (1.18,1.27)	<0.001	*SD*	1.30 (1.26,1.34)	<0.001	1.17 (1.13,1.22)	<0.001
ARV	1.16 (1.14,1.19)	<0.001	1.06 (1.03,1.10)	<0.001	*ARV*	1.13 (1.11,1.16)	<0.001	1.05 (1.02,1.08)	0.002
VIM	1.40 (1.35,1.44)	<0.001	1.23 (1.19,1.28)	<0.001	*VIM*	1.30 (1.27,1.34)	<0.001	1.18 (1.14,1.22)	<0.001
**Pre-eclampsia**					**Pre-eclampsia**				
SD	1.15 (1.11,1.19)	<0.001	1.21 (1.16,1.27)	<0.001	*SD*	1.11 (1.07,1.15)	<0.001	1.18 (1.12,1.23)	<0.001
ARV	1.03 (1.00,1.06)	0.087	1.06 (1.01,1.10)	0.012	*ARV*	1.02 (0.99,1.06)	0.143	1.06 (1.01,1.10)	0.011
VIM	1.15 (1.11,1.19)	<0.001	1.22 (1.16,1.28)	<0.001	*VIM*	1.11 (1.07,1.15)	<0.001	1.19 (1.23,1.24)	<0.001
**PTB**					**PTB**				
SD	1.01 (0.98,1.04)	0.473	1.07 (1.04,1.11)	<0.001	*SD*	0.99 (0.97,1.02)	0.534	1.05 (1.01,1.09)	0.005
ARV	1.01 (0.99,1.04)	0.211	1.03 (1.01,1.09)	<0.001	*ARV*	1.01 (0.99,1.03)	0.492	1.05 (1.02,1.08)	<0.001
VIM	1.01 (0.98,1.04)	0.493	1.07 (1.04,1.11)	<0.001	*VIM*	0.99 (0.97,1.02)	0.514	1.05 (1.01,1.08)	0.007
**SGA infant**					**SGA infant**				
SD	1.05 (1.03,1.07)	<0.001	1.07 (1.04,1.10)	<0.001	*SD*	1.05 (1.02,1.07)	<0.001	1.05 (1.02,1.08)	<0.001
ARV	1.02 (1.00,1.04)	0.022	1.01 (0.99,1.03)	0.382	*ARV*	1.02 (1.01,1.04)	0.011	1.00 (0.99,1.03)	0.758
VIM	1.05 (1.03,1.07)	<0.001	1.07 (1.04,1.10)	<0.001	*VIM*	1.05 (1.03,1.07)	<0.001	1.06 (1.03,1.09)	<0.001

**NICU admission**					**NICU admission**				
SD	1.04 (1.01,1.07)	0.214	1.03 (1.00,1.06)	0.079	*SD*	1.03 (1.01,1.06)	0.020	1.02 (0.98,1.05)	0.296
ARV	1.02 (1.00,1.04)	0.052	1.02 (1.00,1.05)	0.107	*ARV*	1.02 (1.00,1.03)	<0.001	1.02 (0.99,1.04)	0.174
VIM	1.04 (1.01,1.06)	0.002	1.03 (1.00,1.06)	0.050	*VIM*	1.03 (1.01,1.06)	0.020	1.02 (0.99,1.05)	0.236
**Stillbirth**					**Stillbirth**				
SD	1.09 (0.98,1.21)	0.130	1.01 (0.86,1.19)	0.879	*SD*	1.11 (1.00,1.24)	0.042	0.99 (0.85,1.16)	0.913
ARV	1.06 (0.97,1.16)	0.215	1.06 (0.94,1.19)	0.340	*ARV*	1.08 (0.99,1.17)	0.086	1.07 (0.96,1.20)	0.218
VIM	1.09 (0.98,1.21)	0.133	1.01 (0.86,1.18)	0.914	*VIM*	1.11 (1.00,1.23)	0.043	0.99 (0.85,1.16)	0.906
**Neonatal death**					**Neonatal death**				
SD	1.06 (0.98,1.15)	0.152	0.97 (0.87,1.09)	0.604	*SD*	1.11 (1.03,1.20)	0.008	0.97 (0.86,1.09)	0.578
ARV	1.06 (1.00,1.13)	0.053	1.04 (0.96,1.13)	0.358	*ARV*	1.10 (1.04,1.16)	0.002	1.05 (0.97,1.14)	0.234
VIM	1.06 (0.98,1.15)	0.152	0.97 (0.87,1.09)	0.589	*VIM*	1.11 (1.03,1.19)	0.008	0.97 (0.86,1.09)	0.578
**Four- Week Sensitivity Analysis**					**Six- Week Sensitivity Analysis**				
**Outcomes**	**Systolic BP**	**Wald's Test (p)**	**Diastolic BP**	**Wald's Test (p)**	**Outcomes**	**Systolic BP**	**Wald's Test (p)**	**Diastolic BP**	**Wald's Test (p)**
**Gestational hypertension**					**Gestational hypertension**				
SD	1.09 (1.07,1.11)	<0.001	1.10 (1.07,1.13)	<0.001	*SD*	1.07 (1.05,1.09)	<0.001	1.08 (1.05,1.10)	<0.001
ARV	1.05 (1.03,1.06)	<0.001	1.04 (1.02,1.06)	<0.001	*ARV*	1.04 (1.02,1.05)	<0.001	1.03 (1.01,1.05)	<0.001
VIM	1.09 (1.07,1.11)	<0.001	1.10 (1.07,1.13)	<0.001	*VIM*	1.07 (1.05,1.09)	<0.001	1.08 (1.05,1.10)	<0.001
**Severe hypertension**					**Severe hypertension**				
SD	1.21 (1.78,1.24)	<0.001	1.11 (1.07,1.15)	<0.001	*SD*	1.15 (1.13,1.18)	<0.001	1.07 (1.04,1.11)	<0.001
ARV	1.10 (1.08,1.12)	<0.001	1.03 (1.00,1.06)	0.025	*ARV*	1.09 (1.07,1.11)	<0.001	1.03 (1.01,1.06)	0.010

VIM	1.21 (1.18,1.24)	<0.001	1.11 (1.07,1.15)	<0.001	*VIM*	1.16 (1.13,1.19)	<0.001	1.07 (1.04,1.11)	<0.001
**Pre-eclampsia**					**Pre-eclampsia**				
SD	1.07 (1.03,1.11)	<0.001	1.14 (1.09,1.20)	<0.001	*SD*	1.04 (1.01,1.08)	0.025	1.07 (1.03,1.13)	0.003
ARV	1.03 (1.00,1.06)	0.032	1.06 (1.02,1.10)	0.007	*ARV*	1.03 (1.01,1.06)	0.018	1.04 (1.00,1.08)	0.048
VIM	1.07 (1.03,1.11)	<0.001	1.15 (1.09,1.21)	<0.001	*VIM*	1.04 (1.01,1.08)	0.025	1.08 (1.02,1.13)	0.005
**PTB**					**PTB**				
SD	0.97 (0.95,1.00)	0.052	1.01 (0.97,1.04)	0.725	*SD*	0.98 (0.95,1.00)	0.071	0.99 (0.96,1.02)	0.582
ARV	1.00 (0.98,1.02)	0.942	1.03 (1.01,1.06)	0.018	*ARV*	1.00 (0.99,1.02)	0.698	1.02 (1.00,1.05)	0.074
VIM	0.97 (0.95,1.00)	0.052	1.01 (0.97,1.04)	0.666	*VIM*	0.98 (0.95,1.00)	0.071	0.99 (0.96,1.02)	0.603
**SGA infant**					**SGA infant**				
SD	1.03 (1.01,1.05)	0.004	1.05 (1.02,1.07)	0.001	*SD*	1.02 (1.00,1.04)	0.071	1.01 (0.99,1.04)	0.364
ARV	1.02 (1.00,1.03)	0.038	1.01 (0.99,1.03)	0.430	*ARV*	1.02 (1.00,1.03)	0.024	1.00 (0.98,1.02)	0.740
VIM	1.03 (1.01,1.05)	0.005	1.04 (1.01,1.07)	0.003	*VIM*	1.02 (1.00,1.04)	0.094	1.01 (0.98,1.04)	0.448
**NICU admission**					**NICU admission**				
SD	1.01 (0.99,1.04)	0.434	0.99 (0.96,1.03)	0.585	*SD*	1.01 (0.98,1.03)	0.628	0.98 (0.95,1.01)	0.204
ARV	1.01 (0.99,1.03)	0.366	1.00 (0.98,1.03)	0.792	*ARV*	1.01 (0.99,1.03)	0.198	0.99 (0.97,1.02)	0.528
VIM	1.01 (0.98,1.04)	0.480	1.00 (0.97,1.03)	0.929	*VIM*	1.01 (0.98,1.03)	0.652	0.98 (0.95,1.01)	0.217
**Stillbirth**					**Stillbirth**				
SD	1.05 (0.94,1.18)	0.355	1.02 (0.88,1.20)	0.770	*SD*	1.08 (0.97,1.20)	0.166	1.03 (0.89,1.20)	0.676
ARV	1.04 (0.96,1.13)	0.365	1.11 (1.00,1.23)	0.057	*ARV*	1.04 (0.96,1.13)	0.314	1.09 (0.99,1.21)	0.082
VIM	1.05 (0.94,1.18)	0.359	1.02 (0.88,1.19)	0.802	*VIM*	1.08 (0.97,1.19)	0.171	1.02 (0.88,1.18)	0.786
**Perinatal death**					**Perinatal death**				
SD	1.07 (0.99,1.15)	0.105	0.98 (0.88,1.09)	0.691	*SD*	1.07 (0.98,1.15)	0.127	0.97 (0.87,1.08)	0.558
ARV	1.07 (1.01,1.13)	0.020	1.06 (0.99,1.15)	0.110	*ARV*	1.07 (1.01,1.13)	0.019	1.09 (0.99,1.21)	0.082

VIM	1.07 (0.99,1.15)	0.110	0.98 (0.88,1.09)	0.681	*VIM*	1.06 (0.98,1.14)	0.139	0.97 (0.87,1.08)	0.557

ARV indicates average real variability; BP, blood pressure; dBP, diastolic blood pressure; OR, odds ratio; NICU, neonatal intensive care unit; PTB, preterm birth; sBP, systolic blood pressure; SD; standard deviation; SGA, small-for-gestational-age; VIM, variability independent of the mean.

Data are adjusted OR (95% CI) (odds ratio (95% confidence interval). ORs are adjusted for maternal age, maternal BMI, parity, and use of antihypertensives.

Cells in which 95% CI do not overlap 1 are highlighted in green.

One-, two-, four-, and six-week analyses are SD and ARV (for both sBP and dBP) calculated excluding BP values 7, 14, 28 and 42 days from delivery respectively.

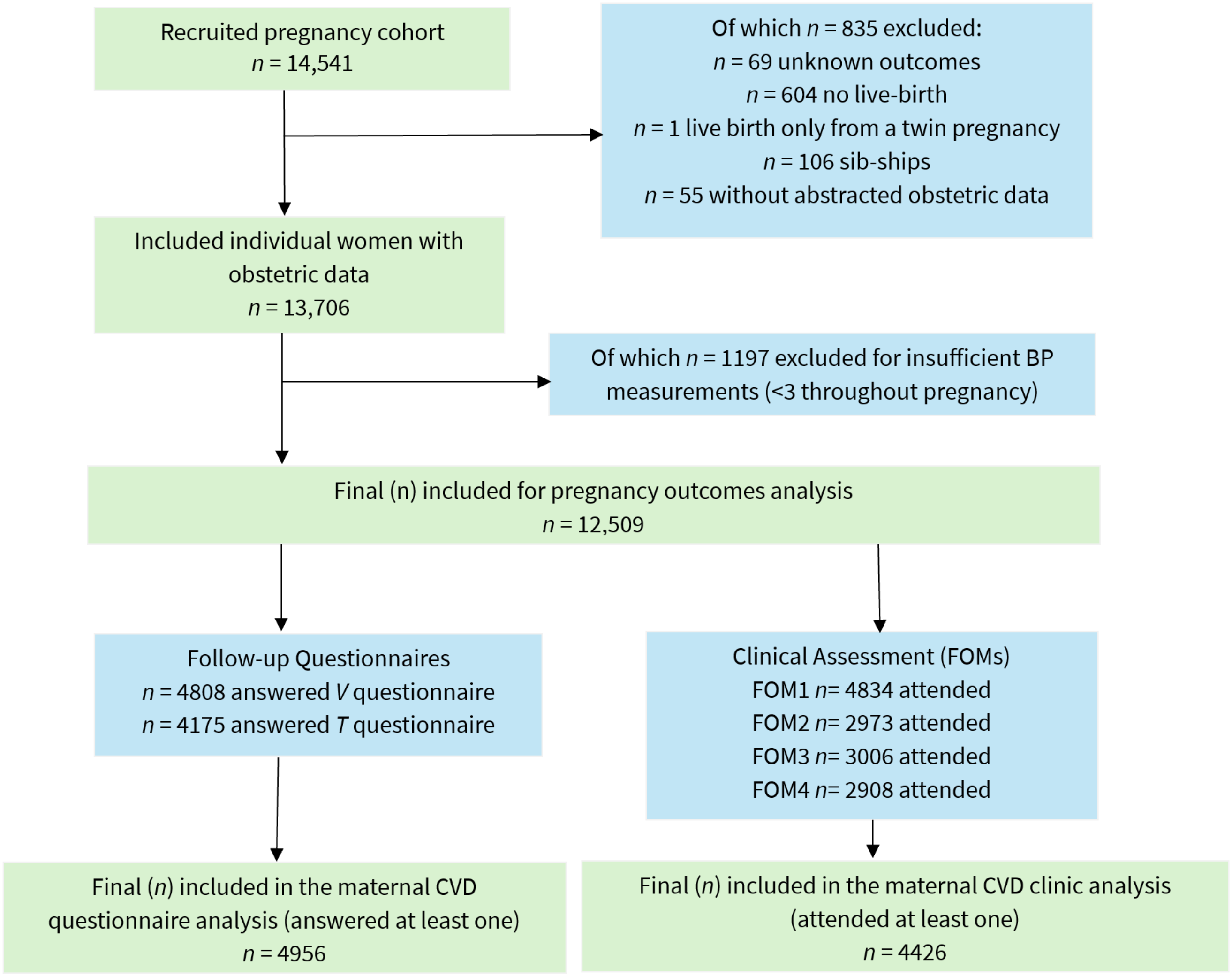

Figure S1. Participant Inclusion Flow Diagram

ALSPAC indicates Avon Longitudinal Study of Parents and Children; BP, blood pressure; CVD, cardiovascular disease; FOMs, follow-up mothers.
